# Editorial: A hill of needs: Non-HLA antibodies and transplantation

**DOI:** 10.3389/fimmu.2022.1005129

**Published:** 2022-08-22

**Authors:** H.G. Otten, Mélanie Dieudé, Michelle J. Hickey

**Affiliations:** ^1^ Central Diagnostic Laboratory (CDL)/Center for Translational Immunology (CTI), University Medical Center, Utrecht, Netherlands; ^2^ Research Centre, Centre hospitalier de l’Université de Montréal (CRCHUM), Montréal, QC, Canada; ^3^ Université de Montréal, Montréal, QC, Canada; ^4^ Héma-Québec, Montréal, QC, Canada; ^5^ Canadian Donation and Transplantation Research Program, Edmonton, AB, Canada; ^6^ UCLA Immunogenetics Center, Department of Pathology and Laboratory Medicine, University of California Los Angeles, David Geffen School of Medicine, Los Angeles, CA, United States

**Keywords:** Non-HLA, antibody, transplant, antigen, rejection

In recent years a growing body of evidence suggests a relationship between the presence of non-HLA antibodies and graft loss and/or rejection episodes after organ transplant (1–3). Non-HLA antibodies are divided into those with specificity for alloantigens, such as the polymorphic alloantigen major histocompatibility class-1 chain A (MICA), and those with specificity for a range of autoantigens such as the angiotensin II type 1 receptor (AT1R), K-alpha 1 tubulin, or cardiac myosin. But, the diversity in form and function of non-HLA antibodies extends beyond this initial distinction. Non-HLA antibodies recognize antigens found in various cellular compartments including the cell membrane, intracellular spaces, and secreted extracellular vesicles, and further, the formation and likely the function of non-HLA antibodies is multimechanistic.

While, the data indicate that detection of non-HLA antibodies may be useful in identifying patients at risk for allograft injury or graft loss in organ specific contexts, thus far no human studies have proven that non-HLA antibodies can directly contribute to the pathogenesis of graft dysfunction. Non-HLA antibodies can be detected using commercial reagents or with laboratory-developed tests. However, lacking are reference sera containing defined concentrations of non-HLA antibodies, and therefore, the definition of a positive result may be based on thresholds that lack clinical relevance. The combination of these factors lead to technical variation between studies, and as such there is no one standard assay in the field that defines the impact of non-HLA antibodies on graft loss. A complete overview on the detection of non-HLA antibodies by solid-phase (ELISA, Luminex) and cell-based (culture, flow-cytometry) techniques is provided by the state-of-the–art review written by Lammerts et al..

Luminex analysis was performed by Senev et al. in a large study including pre- and post-transplant sera (n=2870 from 874 patients), which were retrospectively tested for the presence of 82 different non-HLA antibodies. From the results it was clear that patients having a broad sensitization against non-HLA targets are associated with an increased risk of ABMR histology after kidney transplantations in the absence of HLA-DSA. One application of cell-based assays was provided by the group from Lammerts et al. describing a rare case of hyperacute rejection in the absence of detectable donor-specific HLA antibodies. Crossmatch assays using patient serum showed antibody binding and complement-dependent-cytotoxicity of primary renal endothelial cells, without reactivity to lymphocytes. Removal of these antibodies allowed a successful subsequent kidney transplant. A further development of this cell-based assay employs endothelial cells devoid of surface class-I and –II expression allowing selective detection of non-HLA-antibodies without interference from HLA antibodies (4).

Formation of non-HLA antibodies, whether preformed at transplant or formed *de novo* appears to be unlike classical allo-sensitization to HLA antigens, and is multimechanistic and seemingly associated with various tissue environmental contexts (organ specific and heterogeneous tissue antigen expression, ischemia reperfusion injury, microvascular injury, bronchial obliterans syndrome). An increasing body of literature summarized by Ravichandran et al. suggests that extracellular vesicles (EVs) released from donor organs or from the recipient damaged tissues pre- peri- and post-transplant may act as an accelerator of rejection. Several reports identify immunomodulatory function for theses vesicles including the potency to trigger autoimmune responses and the production of non-HLA autoantibodies. It will be crucial to further define the role and function of the tissue microenvironment and circulating EVs as effectors or biomarkers along with non-HLA antibodies- particularly to identify transplant recipients at risk for developing chronic rejection.

The functionality of non-HLA antibodies as well as the putative association or causation with graft injury and loss is likely to also be divergent between antibody specificities. While some non-HLA autoantibodies appear to be low affinity polyspecific, epitope level specificities, the impact of inflammation on their production and affinity maturation as well as their Fc-mediated effector functions are still largely unexplored. Thus far, regarding the array of non-HLA antibodies that have been identified through multiple cell-based and solid phase testing modalities, basic mechanistic study is limited to only a few specificities, *eg*. collagenV, and perlecan (LG3). These bodies of work, and others are summarized in exceptionally researched reviews by Ravichandran et al., and Lebraud et al., highlighting the immunobiological processes underlying the generation and function of certain non-HLA antibodies in certain transplant contexts. And still, an uphill journey to understanding the clinical utility of non-HLA antibodies remains.

## Author contributions

All authors listed have made a substantial, direct, and intellectual contribution to the work and approved it for publication.

## Conflict of interest

MH receives a share of royalty received on sales of the Lifecodes Non-HLA Antibody kit, managed by UCLA.

The remaining authors declare that the research was conducted in the absence of any commercial or financial relationships that could be construed as a potential conflict of interest.

## Publisher’s note

All claims expressed in this article are solely those of the authors and do not necessarily represent those of their affiliated organizations, or those of the publisher, the editors and the reviewers. Any product that may be evaluated in this article, or claim that may be made by its manufacturer, is not guaranteed or endorsed by the publisher.
